# Supplementary material for: End Sequence Analysis Toolkit (ESAT) expands the extractable information from single-cell RNA-seq data
Source: Genome Res. 2016 Oct;26(10):1397–410. doi: 10.1101/gr.207902.116 (PMC5052061; doi:10.1101/gr.207902.116)
Supplement: Supplemental Material [file supp_gr.207902.116_Supp_Materials.tar.gz › ESAT-esat_v0.1/doc/html/interfaceumms_1_1core_1_1model_1_1score_1_1_window_score.html]

ESAT: umms.core.model.score.WindowScore Interface Reference


ESAT


- Main Page
- Related Pages
- Packages
- Classes
- Files

- Class List
- Class Index
- Class Hierarchy
- Class Members

All Classes Namespaces Files Functions Variables Enumerator Pages

- umms
- core
- model
- score
- WindowScore

Classes |
Public Member Functions |
List of all members

umms.core.model.score.WindowScore Interface Reference

Inheritance diagram for umms.core.model.score.WindowScore:

[legend]

|  |  |
| --- | --- |
| Classes | |
| class | AbstractWindowScore |
|  | |

|  |  |
| --- | --- |
| Public Member Functions | |
| Annotation | getAnnotation () |
|  | |
| double | getScore () |
|  | |

## Detailed Description

Represents a window scoring object. All of the code for calculating scores should be included in implementers of this class

## Member Function Documentation

|  |  |  |  |  |
| --- | --- | --- | --- | --- |
| Annotation umms.core.model.score.WindowScore.getAnnotation | ( |  | ) |  |

Returns the underlying window

Implemented in umms.core.model.score.WindowScore.AbstractWindowScore.

|  |  |  |  |  |
| --- | --- | --- | --- | --- |
| double umms.core.model.score.WindowScore.getScore | ( |  | ) |  |

Returns the number of reads overlapping this region

Returns
:   number of reds overlapping this window Returns the "score" as defined by each subclass (e.g. count, ratio, sum of annotation scores, etc.)

Implemented in umms.core.model.score.CountScore, umms.core.model.score.WindowAllScore, umms.core.model.score.RatioScore, umms.core.model.score.NewRatioScore, umms.core.model.score.SumScore, umms.core.model.score.EndInWindowCountScore, umms.core.model.score.StartInWindowCountScore, and umms.core.model.score.AbstractListScore.

---

The documentation for this interface was generated from the following file:

- java/umms/core/model/score/WindowScore.java


---

Generated on Fri Aug 15 2014 15:11:14 for ESAT by  

 1.8.7
